# Elucidating the Unique J-Shaped Protomer Structure of Amyloid-β(1-40) Fibril with Cryo-Electron Microscopy

**DOI:** 10.3390/ijms26031179

**Published:** 2025-01-29

**Authors:** Raymond N. Burton-Smith, Maho Yagi-Utsumi, Saeko Yanaka, Chihong Song, Kazuyoshi Murata, Koichi Kato

**Affiliations:** 1 Exploratory Research Center on Life and Living Systems, National Institutes of Natural Sciences, Okazaki 444-8787, Japanmahoyagi@ims.ac.jp (M.Y.-U.); saeko-yanaka@ims.ac.jp (S.Y.); chsong@pusan.ac.kr (C.S.); 2 National Institute for Physiological Sciences, National Institutes of Natural Sciences, Okazaki 444-8585, Japan; 3 Graduate Institute for Advanced Studies, SOKENDAI, Kanagawa 240-0193, Japan; 4 Institute for Molecular Science, National Institutes of Natural Sciences, Okazaki 444-8787, Japan; 5 Graduate School of Pharmaceutical Sciences, Nagoya City University, Nagoya 467-8603, Japan

**Keywords:** amyloid-β, Alzheimer’s disease, fibril, cryo-electron microscopy

## Abstract

Although the structural diversity of amyloid-β (Aβ) fibrils plays a critical role in the pathology of Alzheimer’s disease (AD), the mechanisms underlying this diversity remain poorly understood. In this study, we report the discovery of a novel J-shaped protomer structure of Aβ40 fibrils, resolved at 3.3 Å resolution using cryo-electron microscopy. Under controlled conditions (20 mM sodium phosphate buffer, pH 8.0) designed to emphasize intra-protomer interactions and slow fibril elongation, the J-shaped structure revealed distinct salt bridges (e.g., D1-K28, R5-E22) that stabilize the fibril core. These findings expand our understanding of the free energy landscape of fibril formation, shedding light on how specific environmental factors, such as pH and ionic strength, may influence fibril polymorphism. Importantly, the unique features of the J-shaped protomer provide insights into the structural basis of amyloid plaque diversity in AD and suggest potential therapeutic strategies targeting intra-protomer interactions. This study underscores the importance of fibril polymorphism in AD pathology and offers a foundation for future research into fibril-targeted therapies.

## 1. Introduction

The pathology of Alzheimer’s disease (AD) is primarily characterized by the fibrillization and deposition of amyloid-β (Aβ) in the brain [1,2]. Aβ is generated through the cleavage of a precursor membrane protein by β-secretase and γ-secretase, resulting in the production of the Aβ40 and Aβ42 isoforms [3]. Therefore, inhibiting Aβ polymerization and removing Aβ fibrils are promising therapeutic strategies for Alzheimer’s disease, raising the need for detailed information on the fibril structure of Aβ for their advancement [4,5,6].

Early studies using X-ray diffraction and the following solid-state nuclear magnetic resonance (ssNMR) have revealed a common structural characteristic in Aβ fibrils: they typically consist of cross-β structures comprising two or more protofilaments, each featuring extended β-sheets running parallel to the direction of fibril growth [7]. ssNMR studies have indicated that U-shaped protomers are symmetrically arranged in Aβ40 fibrils [8], while Aβ42 fibrils exhibit protomers adopting an S-shaped conformation, with the C-terminal segment actively engaging in intra-molecular interactions to stabilize the fibril structure [9].

Recent advancements in cryo-electron microscopy (cryo-EM) have allowed for the visualization of Aβ fibril structures, primarily utilizing fibrils isolated from the brain tissues of AD patients as seeds to catalyze the polymerization of monomeric Aβ under controlled laboratory conditions [10,11,12]. The accumulation of structural data has revealed that environmental factors, likely contingent on accumulation sites within the brain, can determine fibril morphology [13]. Moreover, the morphology of Aβ fibrils prepared in vitro can be influenced by various extrinsic conditions, such as ionic strength, pH, temperature, pressure, agitation, and gravity [14,15,16,17].

Particularly, Aβ40 fibrils exhibit considerable variability in protomer conformation and consequent fibril morphology, likely due to the lack of C-terminal stabilizing interactions compared to Aβ42 fibrils. Cryo-EM studies of Aβ40 fibrils have provided two distinct classes of protomer structures besides U-shaped structures [8]: C-shaped [11] and I-shaped [10].

The varying protomer conformations, characterized by intra-protomer side-chain interactions, can be stabilized or trapped through inter-protomer interactions during fibril formation. In essence, fibril morphology is determined by the balance between intra- and inter-protomer interactions, depending on various intrinsic and extrinsic factors. Most structural studies of Aβ40 fibrils have been conducted under conditions that facilitate rapid fibrillization, such as agitation, sonication, or the addition of fibril seeds. Consequently, the structural diversity of Aβ fibrils formed under conditions that slow fibril elongation remains poorly understood. To address this gap, we aimed to explore the free energy landscape of Aβ40 fibril formation by characterizing fibrils grown under controlled conditions suppressing inter-protomer interactions.

Quantitative kinetic analyses have demonstrated the significant impacts of pH and ionic strength modulation on Aβ fibril formation [18,19]. As Aβ carries a net negative charge, it naturally repels other Aβ monomers or fibrils via electrostatic repulsion. Nevertheless, the addition of salt effectively counteracts this repulsion, facilitating fibril formation. In accordance with this principle, low ionic strength (20 mM sodium phosphate) and high pH (8.0) conditions were specifically chosen in this study in order to enhance inter-protomer electrostatic repulsion, thus facilitating intra-protomer interactions over protomer assembly. These Aβ40 fibrils, prepared under slow growth conditions, were subjected to cryo-EM analysis, and their characteristics were compared with previously reported structures. Through this approach, this study provides insights into the molecular mechanisms underlying fibril polymorphism, which may have implications for understanding the pathology of Alzheimer’s disease and its therapeutic targets.

## 2. Results

### 2.1. Kinetics of Aβ Fibril Formation

**Figure 1:**
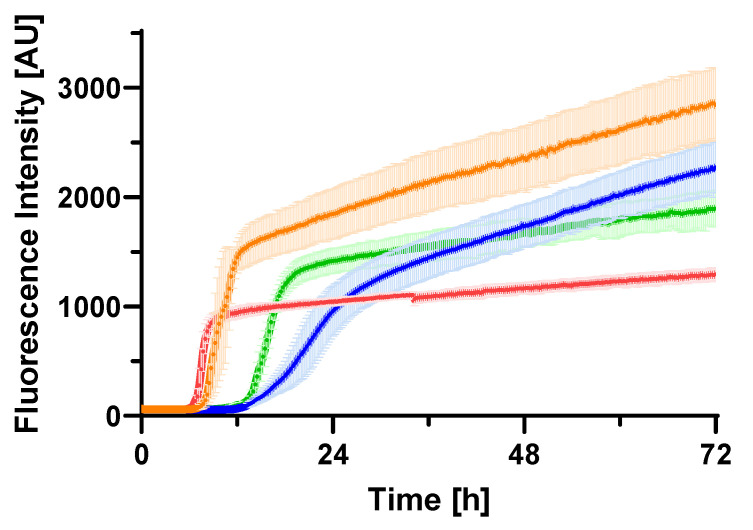
ThT fluorescence assay of Aβ40. Aβ40 solutions (0.1 mM) were prepared in sodium phosphate buffers and incubated at 37 °C. The buffers used were 100 mM pH 7.4 (red), 100 mM pH 8.0 (orange), 20 mM pH 7.4 (green), and 20 mM pH 8.0 (blue). Each intensity value represents the mean ± SD of the three values.

In previous cryo-EM studies focusing on Aβ40 fibrils, one of the mildest conditions involved the absence of seed addition or agitation, utilizing a 100 mM phosphate buffer at pH 7.4. To compare the time course of fibril formation, we probed the process using thioflavin T (ThT) fluorescence enhancement with higher pH and/or lower ionic salt concentrations. This comparative analysis confirmed that Aβ fibrillization proceeds considerably slower in a 20 mM sodium phosphate buffer at pH 8.0 (Figure 1). Compared to the rapid fibril elongation observed in 100 mM sodium phosphate buffer at pH 7.4, the slower elongation rate in 20 mM sodium phosphate buffer at pH 8.0 offered a unique opportunity for structural analysis. These slower conditions facilitated the detailed examination of intra-protomer interactions and the structural organization of fibrils.

### 2.2. J-Shaped Protomer Structure of Aβ Fibrils Revealed by Cryo-EM

**Figure 2:**
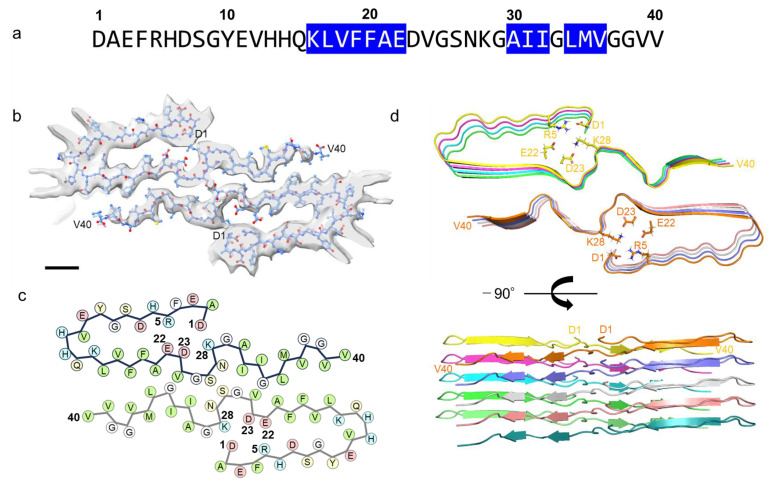
Cryo-EM structure of J-shaped Aβ40 fibrils: (**a**) Sequence of Aβ40, with the observed three β-strands colored blue. (**b**) Top view of the cryo-EM density map of Aβ40 fibrils, with the core layer model overlaid. The ball-and-stick model represents the atomic structure of the fibril backbone within the cryo-EM density. The map is displayed at a contour level of 9 σ, with the scale bar indicating 1 nm. (**c**) Packing scheme of one molecular layer of the fibril. (**d**) Ribbon representation of the top (upper) and side views (lower) of the J-shaped Aβ40 fibril core, containing five molecular layers with a dimer.

Aβ40 fibrils prepared under the designed condition were subjected to cryo-EM analysis, resulting in the achievement of a high-resolution cryo-EM structure at 3.3 Å (Figure 2 and Figure S1). A 3D density map unveiled a left-handed cross-β structure formed by two protofibrils, which we had previously confirmed via AFM [17]. Within the fibril core, a twist of 179.2° was observed, accompanied by a helical rise of 2.454 Å, which describes the upward distance that the structure moves along its helical axis with each turn of the helix. Notably, the cross-sectional view (top view) depicted discernible electron density spanning from D1 to V40. By fitting the Aβ40 molecular model into the electron density map, we elucidated the conformational arrangements and interactions of protomers.

### 2.3. Comparative Analysis with Previously Reported Fibril Structures

**Figure 3:**
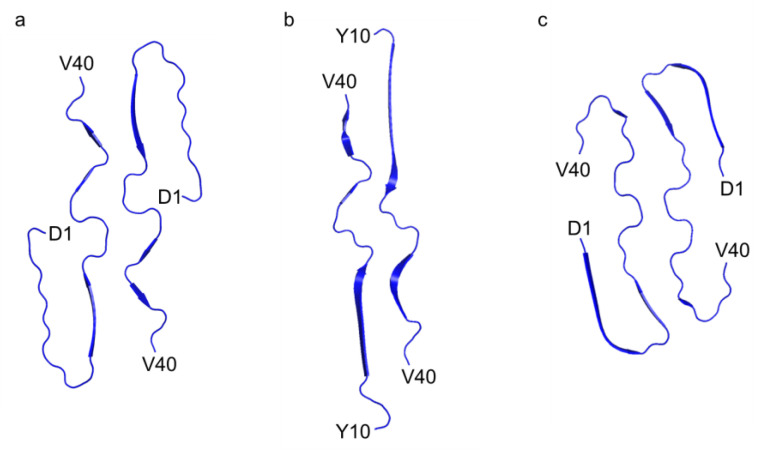
Schematic view of Aβ40 fibril core: (**a**) J-shaped fibril structure (this research, PDB: 9IIO). (**b**) I-shaped fibril structure (PDB: 6W0O). (**c**) C-shaped fibril structure (PDB: 6SHS).

**Figure 4:**
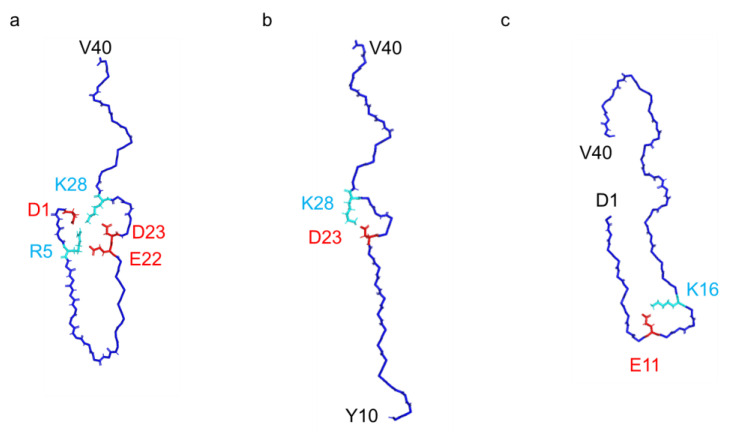
Intra-protomer interactions of Aβ40 fibrils: (**a**) J-shaped fibril structure (this research, PDB: 9IIO). (**b**) I-shaped fibril structure (PDB: 6W0O). (**c**) C-shaped fibril structure (PDB: 6SHS). The side chains of amino acid residues forming salt bridges are displayed in stick representation, with acidic amino acids in red and basic amino acids in cyan.

In contrast to previously documented I-shaped and C-shaped structures [10,11], our fibril structure presented a distinctive J-shaped protomer conformation (Figure 3). Although it bears resemblance to the I-shaped structure, in terms of the extended segment following H13 with discontinuous β-strands, it exhibits unique characteristics. Specifically, in the J-shaped structure, the N-terminal D1–H13 segment folds back against the extended portion of the same protomer (Figure 4). The intra-protomer interaction of the J-shaped fibril is characterized by the formation of salt bridges between D1 and K28, as well as between R5 and E22; meanwhile, in the I-shaped structure, the N-terminal D1–V12 segment lacks order, contributing minimally to the density (Figure S2).

**Figure 5:**
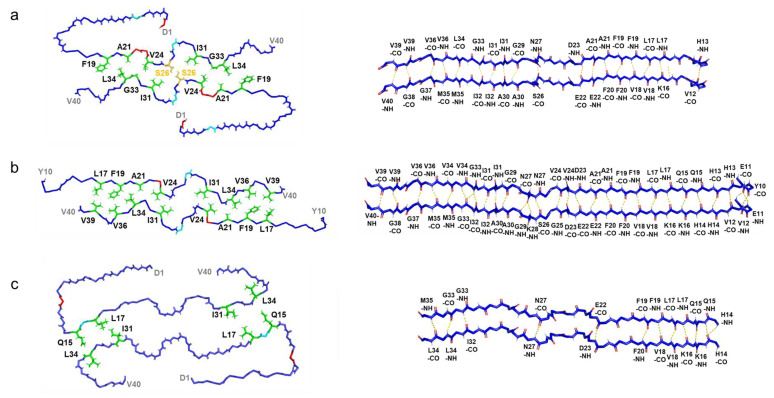
Inter-protomer interactions of Aβ40 fibrils. Cross-sectional view of ((**a**), left) the J-shaped fibril (this research, PDB: 9IIO), ((**b**), left) the I-shaped fibril (PDB: 6W0O), and ((**c**), left) the C-shaped fibril (PDB: 6SHS). Hydrophobic residues involved in inter-protomer interactions are highlighted in green, and residue S26 is marked in yellow. The acidic amino acid residues and basic amino acid residues involved in intra-protomer interactions are shown in red and cyan, respectively. Hydrogen bonding network in ((**a**), right) the segment from V12 to V40 in the β-sheet of the J-shaped fibril, ((**b**), right) from Y10 to V40 in the I-shaped fibril, and ((**c**), right) from H14 to M35 in the C-shaped fibril.

Compared to the I-shaped fibrils, the presence or absence of this intra-protomer interaction elicits a distinctive twist in the V24–G29 segment, orchestrated by a minimal number of hydrogen bonds formed between S26–CO and N27–NH within the β-sheet extension (Figure 5), and our fibril structure exhibits notable discrepancies in the interaction mode between two protofilaments. While both I- and J-shaped structures feature an arrangement of antiparallel face-to-face interactions through the segment following Q15, each segment in the J-shaped structure adopts a twisted arrangement with fewer hydrophobic contacts. Moreover, a unique interaction emerges between the S26-OH group at the center of each segment in our J-shaped fibril.

In the C-shaped structure, the N-terminal segment also interacts with the core part, facilitated by a salt bridge between E11 and K16, unlike the J-shaped structure (Figure 4 and Figure S2). As a result, the orientation of the N-terminal segment differs between the C- and J-shaped fibrils. In contrast to the C-shaped fibril, where β-strand formation was observed in the A2–S8 and Y10–H13 regions, the N-terminus remained disordered in our J-shape fibril (Figure 3). At first glance, the recently reported Aβ38 fibrils derived from brain tissues might appear similar to this J-shaped structure; however, this is uncertain due to the absence of the last two C-terminal residues. In fact, the N-terminal region in the Aβ38 fibril forms a salt bridge between E11 and K16, as seen in the C-shaped structure, and the N-terminal fold-back region closely resembles that of the C-shape (Figure S3). Therefore, it is more likely that this Aβ38 fibril would be classified as a C-shaped fibril. Consequently, our J-shaped protomer conformation remains distinct, as evidenced by the unique intra-protomer salt bridge network and the twisted arrangement of the V24–G29 segment.

## 3. Discussion

In our current investigation, we uncovered a distinct J-shaped fibril structure of Aβ40, contrasting with previously reported fibril conformations. Unlike the well-documented C-, I-, or U-shaped structures [8,10,11], this J-shaped conformation highlights an extensive intra-protomer interaction network facilitated by salt bridges formed at D1–R5, D1–K28, R5–E22, and D23–K28 (Figure 4 and Figure S2). Previous studies have typically utilized Aβ40 fibrils prepared via agitation and/or the addition of fibril seeds obtained from the brain tissues of AD patients, which accelerate fibril formation. However, recently reported I-shaped structures have been determined using Aβ40 fibrils grown in a 100 mM phosphate buffer at pH 7.4, under static conditions without stirring or seeds [12].

As the pH rises, the net charge of Aβ40 becomes increasingly negative, leading to heightened inter-molecular electrostatic repulsion, especially under lower ionic strength. To counteract this effect, we employed static incubation in a 20 mM sodium phosphate buffer at pH 8.0, creating an environment conducive to slower fibril growth. This condition allows for the suppression of Aβ40 assembly, thereby emphasizing intrinsic intra-molecular interactions, which contribute to the conformational organization of the protomer prior to assembly. While our data suggest that intra-protomer salt bridges are central to the formation of the J-shaped structure, other factors may also play a role. For instance, small changes in ionic strength or pH could affect the balance of intra- and inter-protomer interactions. Additionally, variations in fibril assembly pathways might stabilize different structural forms, including the J-shaped fibril. Notably, the enhancement of intra-molecular salt bridge formation under low ionic strength is consistent with the findings of this study.

The formation of intra-molecular salt bridges plays a critical role in the conformational pre-organization of Aβ, which dictates subsequent fibrillization [20]. Several familial mutants of Aβ, such as E22G and D23N, involve single amino acid substitutions that reduce the negative charge. These substitutions hinder the conformational stabilization of protomers by interfering with salt bridge formation, as well as promoting Aβ assembly due to weakened electrostatic repulsion. To comprehensively characterize Aβ fibril formation considering such mutational effects, controlling conformational pre-organization and assembly processes is crucial. Our previous studies have demonstrated that Aβ assembly slows down under microgravity conditions, offering a promising approach for exploring the free energy landscape of Aβ fibril formation [17].

From a translational perspective, this study highlights potential therapeutic targets, particularly by identifying intra-protomer interactions that stabilize the J-shaped conformation. These interactions, such as the D1–K28 and R5–E22 salt bridges, could serve as potential sites for therapeutic intervention. While this study provides a detailed characterization of J-shaped conformation, it is important to acknowledge the in vitro nature of the experiments, which may not fully reflect the physiological environment. Future research should aim to validate these findings under physiological conditions, including exploring the relevance of J-shaped conformation in vivo. Investigating the effects of disease-relevant mutations—particularly those that influence electrostatic interactions—could further enhance our understanding of Aβ fibrillization and its pathological implications.

## 4. Materials and Methods

### 4.1. ThT Assay

Custom-synthesized Aβ40 was purchased from Toray Research Chemicals Co., Ltd. (Tokyo, Japan) Aβ40 powder was dissolved in 0.1% (*v*/*v*) ammonia solution to a concentration of 5 mM and then diluted to 0.1 mM using 20 mM or 100 mM sodium phosphate buffer (pH 7.4 or 8.0) containing 0.2 mM ethylenediaminetetraacetic acid (EDTA). During this process, a 2 mM ThT solution was added to achieve a final concentration of 0.2 mM. Each sample was dispensed into multiple wells of a 96-well half-area, low-binding polyethylene glycol coating plate (Corning 3881, Corning, NY, USA) with a clear bottom, at 100 μL per well.

The kinetic assays were initiated by incubating the 96-well plate at 37 °C under quiescent conditions in a plate reader (Infinite 200Pro, TECAN Japan Co., Ltd., Kawasaki, Japan). ThT fluorescence was measured through the bottom of the plate at excitation and emission wavelengths of 446 nm and 490 nm, respectively, and monitored for three repeats of each sample.

### 4.2. Sample Preparation for Cryo-EM Analyses

Aβ40 dissolved at a concentration of 2 mM in 0.1% (*v*/*v*) ammonia solution was diluted to a concentration of 0.1 mM with 20 mM sodium phosphate buffer (pH 8.0) containing 0.2 mM EDTA. These conditions provide a model that allows for the study of fibril formation dynamics under slowed assembly, complementing structural studies conducted under rapid fibril-forming conditions (e.g., 100 mM sodium phosphate, pH 7.4). Each sample was dispensed at 100 μL per well into multiple wells (0.2 mL each) of a 96-well PCR plate (semi-skirted with upstand, 4titue, Ltd., Birmingham, UK). The top opening of the plate was sealed with a lid (Strips of 8 Flat Optical Caps, 4titude, Ltd.) and covered with Kapton tape (Nitto, Inc., Osaka City, Japan). Samples were prepared on ice to prevent the formation of amyloid fibrils. The sample solutions were frozen at −30 °C for 3 h, followed by freezing at −80 °C until use. The solution was defrosted in a fridge for 16 h at 4 °C and then used at 37 ± 0.5 °C to initiate amyloid fibril formation. After 9 days of incubation, the samples were taken out from the incubator and immediately frozen at −80 °C to prevent further fibril formation. They were kept at this temperature until immediately before cryo-EM analyses.

### 4.3. Cryo-EM Grid Preparation

The Aβ40 amyloid fibrils (0.1 mM) were diluted five times with 20 mM sodium phosphate buffer (pH 8.0) containing 0.2 mM EDTA and subjected to cryo-EM. A 2.5 μL aliquot was placed on a Quantifoil R 1.2/1.3 grid (Quantifoil Micro Tools, Großlobichau, Germany) pre-treated with a glow discharge. The plunge-freezing of the specimen was performed at 4 °C and 95% humidity using a Vitrobot Mark-IV (Thermo Fisher Scientific, Waltham, MA, USA). The frozen grids were kept in a cryo-storage under liquid nitrogen until use.

### 4.4. Cryo-EM Data Acquisition

Micrograph movies were acquired using a Cs-corrected Titan Krios (Thermo Fisher Scientific, USA) equipped with a Gatan K3 direct electron detector (Gatan Inc., Pleasanton, CA, USA) at the Institute for Protein Research, Osaka University. Spherical aberration after Cs correction was 0.027 mm. A total of 58 frames were recorded for each movie under low-dose conditions, with a total dose of 50 e^−^/Å^2^. Three grids were used for data acquisition. A total of 7483 micrographs were collected during a single acquisition period at 64,000× magnification, equivalent to an effective pixel size of 1.14 Å. Micrographs were acquired with an underfocus ranging from 0.8 to 2 μm. Data collection and image processing information are summarized in Supplementary Table S1.

### 4.5. Cryo-EM Data Analysis

The micrograph movies were split into 8 groups, and individual gain references were generated using the “sum_all_tiff_files” module of cisTEM version 1 [21]. Each group was imported into RELION 3.1 [22] and motion-corrected with MotionCor2 (1.3.1) [23] individually before all subsets were combined, following which CTF estimation was carried out using CTFFIND 4.1.14 [24]. After removing micrographs containing no fibrils, aggregated fibrils, or poor ice conditions, a total of 3111 micrographs remained. Fibrils were manually picked with the RELION filament picker (RELION 3.1) [25] and extracted with an estimated helical rise of 4.8 Å and 5 asymmetric units in each segment, resulting in a total of 1,292,904 segments. These segments were passed to 2D classification, where they were classified into 150 classes with the “Ignore CTF to first peak” option enabled. Clear fibril classes, comprising 771,375 segments, were then selected and passed through another round of 2D classification into 120 classes. After this, clear classes containing 681,051 segments were selected once again. Class averages were passed to “relion_helix_inimodel2d”, and an initial model was generated at 8 Å. Different 3D classifications were carried out, experimenting with the imposition of C1 and C2 symmetry. Finally, C1 symmetry with a “pseudo-2(1)-screw”, similar to the structure reported by Ghosh et al. [10], was used. Helical symmetry searches resolved a class containing 577,094 segments with a helical symmetry of 179.2° twist and 2.454 Å rise. Then, 3D refinement with iterative CTF refinement and a single round of Bayesian particle polishing was carried out using default parameters, resulting in a final reconstruction with a resolution of 3.3 Å.

### 4.6. Visualization

Micrographs, particles, and 2D classes were visualized using the “relion_display” module of RELION 3.1 [26], while 3D maps and PDB models were generated using UCSF Chimera (1.16) [27] or UCSF ChimeraX (1.5) [28].

### 4.7. Model Building

PDB 6W0O was used as a starting point. The missing residues of the N-terminal (ASP1 to GLY9) were added in ChimeraX [28], and ISOLDE [29] was used to roughly fit the fibrils via molecular dynamics. The central serine, SER26, snapped into a “ladder” arrangement between the fibrils. PHENIX 1.20.1 [30] was used for energy minimization and refinement of the final model.

## 5. Conclusions

This study provides a detailed characterization of a novel J-shaped protomer structure of Aβ40 fibrils, offering new insights into the energy landscape governing amyloid fibril formation. By elucidating its unique intra-protomer interactions, our findings enhance the understanding of Aβ polymorphism and its role in Alzheimer’s disease pathology. These results highlight the structural diversity of Aβ fibrils and provide a robust framework for therapeutic strategies targeting specific fibril conformations, such as inhibitors designed to disrupt intra-protomer interactions. Furthermore, future studies should examine the physiological relevance of these findings and investigate how familial mutations influence electrostatic interactions in order to deepen our understanding of Aβ fibrillization.

## Data Availability

Electrostatic potential maps of the Aβ40 amyloid fibril have been deposited in the EMDB with the access code 60603. The PDB model has been deposited in the wwPDB databank with the accession code 9IIO.

## Supplementary Materials

The following supporting information can be downloaded at: https://www.mdpi.com/article/10.3390/ijms26031179/s1.

## Abbreviations

The following abbreviations are used in this manuscript:

AD	Alzheimer’s disease
Aβ	Amyloid-β
AFM	Atomic force microscopy
cryo-EM	Cryo-electron microscopy
EDTA	Ethylenediaminetetraacetic acid
ssNMR	Solid-state nuclear magnetic resonance
ThT	Thioflavin
