# Supplementary material for: Elucidating the Unique J-Shaped Protomer Structure of Amyloid-β(1-40) Fibril with Cryo-Electron Microscopy
Source: Int J Mol Sci. 2025 Jan 29;26(3):1179. doi: 10.3390/ijms26031179 (PMC11817843; doi:10.3390/ijms26031179)
Supplement: Supplementary file 1 [file ijms-26-01179-s001.zip › ijms-3421202-supplementary.pdf]

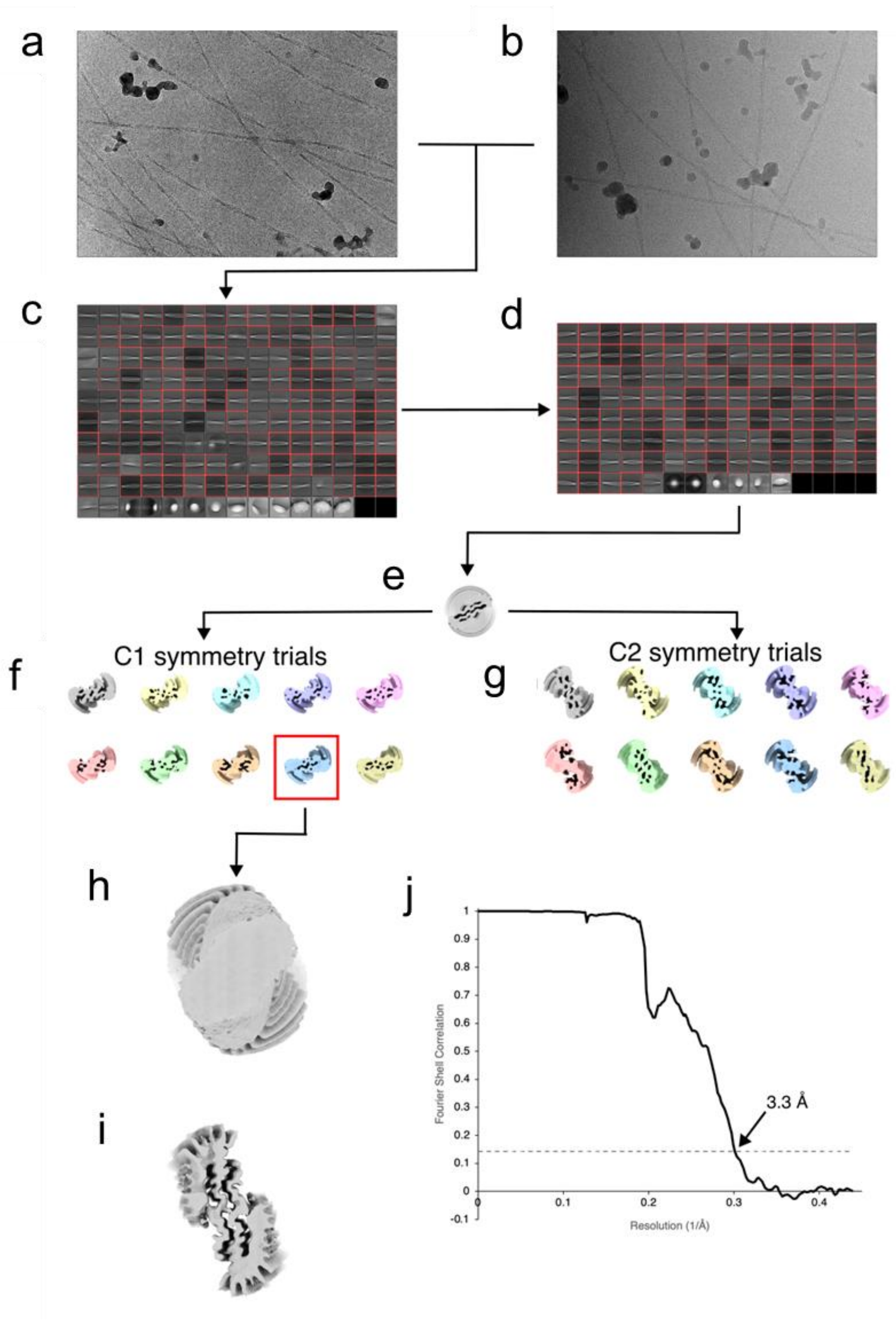

Fig. S1. Cryo-electron microscopy processing flowchart. An overview of the cryo-EM processing workflow. (a) Representative micrograph at higher defocus. (b) Representative micrograph at lower defocus. Fibrils were manually picked, and segments were extracted before a first round of 2D classification (c). After that, high resolution classes were selected and subjected to further 2D classification (d). Good classes were used to generate an initial model (e), which was then used in different 3D classifications with C1 symmetry (f) or C2 symmetry (g) before identification of a class with contiguous density containing 577,094 segments. It was passed to 3D refinement (h). (i) A central view of the map. After cycles of 3D refinement and CTF refinement, a single round of Bayesian polishing was carried out, resulting in a final global resolution of 3.3 Å (j).

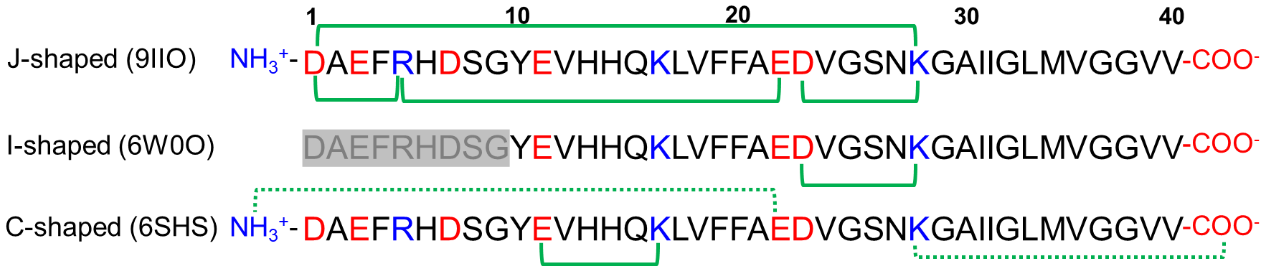

Fig. S2. Schematic representation of salt bridge interactions. Red represents negatively charged residues (Asp and Glu), and blue represents positively charged residues (Arg and Lys). Green lines indicate salt bridge formation between charged residues. The dotted lines represent additional possible salt bridges.

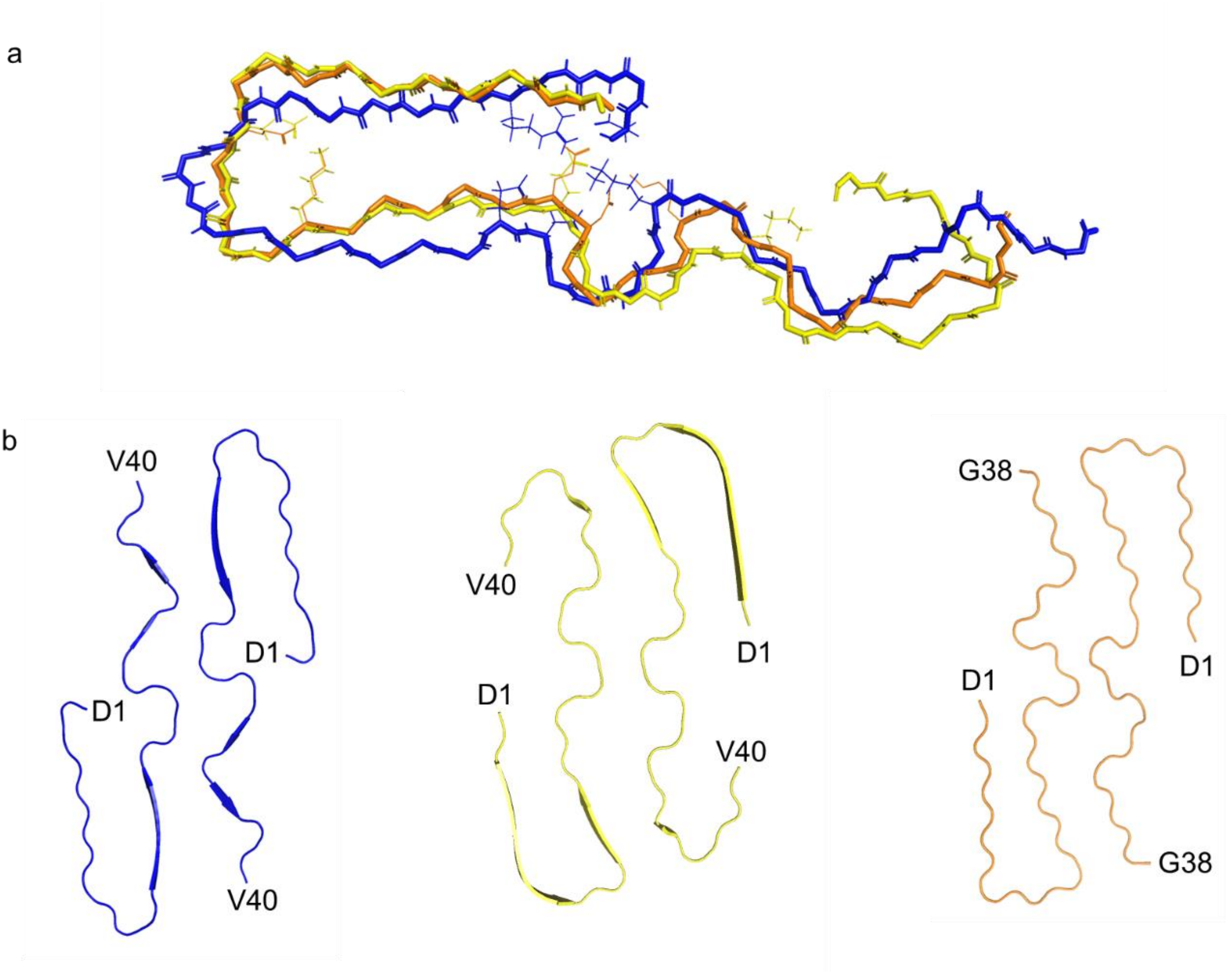

Fig. S3. Structural comparison of Aβ fibrils. (a) Structural overlay of Aβ fibrils from different protomer conformations. The blue, yellow, and orange ribbons represent the J-shaped fibril (this research, PDB: 9IIO), the C-shaped fibril (PDB: 6SHS), and the 'imperfect' C-shaped fibril (PDB: 8QN6), respectively. The RMSD for the overlay of the J-shaped and C-shaped structures was 4.56 Å, while the RMSD for the C-shaped and imperfect C-shaped fibrils was 0.73 Å. (b) Top view of fibril core structure of J-shaped fibril (left), C-shaped fibril (center), and imperfect C-shaped fibril (right).

Tabel S1. Parameters for cryoEM data collection and image processing information

Microscope Parameters	
Model	Titan Krios (Thermo Fisher Scientific)
Acceleration voltage (KV)	300
Spherical aberration (mm)	0.027 (Cs corrected)
Detector	K3 (Gatan)
Magnification	64,000×
Equivalent Å/pixel	1.14
Total dose (e^-/Å^2)	50
Micrographs acquired	7,483
Micrographs accepted	3,111
Total segments extracted	1,292,904
Final segments used	577,094
Model Statistics (PHENIX)	
Chains	40
Atoms	23880 (Hydrogrens: 11640)
Residues	Protein: 1600
Bonds (RMSD)	
Length (Å) (# > 4 σ)	0.003 (0)
Angles (°) (# > 4 σ)	0.683 (0)
MolProbity Score	2.15
Clash score	13.21
Ramachandran plot (%)	
Outliers	0.00
Allowed	9.08
Favoured	90.92
Rotamer outliers (%)	0.00
Cβ outliers	0.00
Peptide plane (%)	
Cis proline/general	0.0/0.0
Twisted proline/general	0.0/0.0
